# Fungal volatile organic compounds show promise as potent molluscicides

**DOI:** 10.1002/ps.5578

**Published:** 2019-10-01

**Authors:** Salim Khoja, Khalifa M Eltayef, Ian Baxter, James C Bull, Edric Joel Loveridge, Tariq Butt

**Affiliations:** ^1^ Department of Biosciences Swansea University Swansea UK; ^2^ Department of Chemistry Swansea University Swansea UK; ^3^ Certis Europe BV Maarssen The Netherlands

**Keywords:** slugs, snails, fungal volatiles, *Metarhizium*, repellents, molluscicides

## Abstract

**BACKGROUND:**

Slugs and snails constitute major crop pests. Withdrawal of metaldehyde has prompted a search for more environmentally friendly yet fast acting molluscicides. This study investigated the response of representative molluscs to conidia and volatile organic compounds (VOCs) of the insect pathogenic fungus *Metarhizium brunneum* Petch.

**RESULTS:**

Conidia of *M. brunneum* had antifeedant/repellent properties with repellency being dependent upon the fungal strain and conidia concentration. Three commonly produced fungal VOCs, 1‐octene, 3‐octanone and 1‐octen‐3‐ol, were repellent at low doses (1–5 μL) but could kill slugs and snails on contact or fumigation. At the highest dose tested (10 μL), 100% mortality was achieved for *Cornu aspersum* Muller (garden snail) and *Derocerus reticulatum* Muller (grey field slug) within 1 h post‐treatment with the first deaths being recorded in <11 min. Aqueous formulations (20% v/v) of the most potent VOCs, 3‐octanone and 1‐octen‐3‐ol, could be sprayed onto plants to kill or drive the pest of the crop with no phytotoxic effects.

**CONCLUSION:**

The sensitivity of terrestrial molluscs to 3‐octanone and 1‐octen‐3‐ol and the ephemeral nature of these compounds makes these excellent candidates for development as mollusc repellents or molluscicides. © 2019 The Authors. *Pest Management Science* published by John Wiley & Sons Ltd on behalf of Society of Chemical Industry.

## Graphical abstract

Slugs and snails avoid plants treated with conidia and VOCs of the insect pathogenic fungus *Metarhizium brunneum*. The VOCs show promise as mollusc repellents or molluscicides.

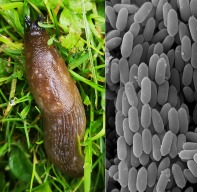

## 1 INTRODUCTION

Snails and slugs are serious pests of agricultural and horticultural crops. They cause feeding damage to both aerial and subterranean parts of the plant, including leaves, shoots, roots, tubers, corms, bulbs, flowers and seed. Besides reducing plant stand and crop yield they increase the risk of infection by opportunistic plant pathogens.^1^ Feeding damage can significantly reduce the aesthetic appearance of plants (e.g. nursery stock, root crops, salads) and hence their marketability. Slug and snail population densities have increased over the years due to changes in crop management such as minimum tillage, direct drilling, and over‐wintering arable crops.^2^, ^3^ Their pest status is set to increase further due to legislative changes to pesticide usage and climate change with weather patterns favoring their population growth.^3^, ^4^ The problem is often exacerbated by the accidental introduction of highly damaging invasive mollusc species such as the golden apple snails (*Pomacea canaliculate* Lamarc*k, Pomacea maculata* Perry*)*, which have devastated rice crops in Asia and pose a serious threat to food security in the region.^5^, ^6^, ^7^

Current mollusc control is still heavily dependent upon the use of chemical pesticides such as metaldehyde, ferric phosphate, methiocarb and thiodicarb.^8^ Metaldehyde‐based products by far dominate the molluscicide bait market worldwide but have corresponding ecotoxicological effects on non‐target species and lead to contamination of drinking water.^5^, ^6^ However, metaldehyde will be withdrawn in the UK by 2020 in order to protect wildlife and to comply with the EC drinking water Directive 98/83/EC.^9^ The nematode *Phasmarhabditis hermaphrodita* Schneider has been developed as a biological molluscicide but is considered expensive and ineffective against mature molluscs.^10^, ^11^ Live nematodes have been shown to elicit avoidance behavior in some slug species.^12^

Various plant‐derived products (e.g. caffeine, neem, terpenoids, isothiocyanates) show promise as molluscicides and mollusc repellents.^13^, ^14^, ^15^, ^16^, ^17^ Whereas some compounds act as feeding deterrents others exhibit fumigant or contact toxicity.^13^, ^17^, ^18^ Although many plant species have been screened to identify potential molluscicides very little attention has been given to fungi as a source of such compounds. Of the limited number of studies, it is clear that slugs avoid or taste and reject the mushroom sporocarp. Wood *et al*.,^19^ investigated the feeding behavior of the banana slug, *Ariolimax columbianus* Gould, on the sweetbread mushroom, *Clitopilus prunulus* Scop, and discovered that 1‐octen‐3‐ol released by this mushroom acted as an antifeedant.

Many fungal species produce low molecular weight volatile organic compounds (VOCs) with insect behavior modifying properties.^20^ Some compounds clearly possess pesticidal or repellent properties.^21^, ^22^, ^23^ Most notable are 1‐octen‐3‐ol, 3‐octanone and 1‐octene, which are produced by a wide range of fungi including saprophytic molds, mushrooms and entomopathogenic fungi including species of *Beauveria* and *Metarhizium*.^24^, ^25^, ^26^, ^27^, ^28^ Exactly why entomopathogenic fungi produce these compounds is unclear but the fact that so many insects exhibit avoidance behavior suggest that they are responding to VOCs.^20^, ^29^ This paper reports that these compounds are highly repellent or toxic to slugs and snails. The potential for development of these ubiquitous volatiles as *novo* molluscicides is discussed.

## 2 METHODS

### 2.1 Collection and maintenance of molluscs

The following slugs and snails were collected from local gardens and parks in Swansea: garden snail (*Cornu aspersum*), great grey or leopard slug (*Limax maximus* Linnaeus), black slug (*Arion ater* Linnaeus), large red slug (*Arion rufus* Linnaeus), grey field slug (*Derocerus reticulatum)* and Yellow slug (*Limax flavus* Linnaeus). The animals were collected at dawn or early evening and maintained in ventilated plastic containers at room temperature (21 ± 2°C) until required. They were fed diverse plant materials (turf, flowers, lettuce leaves) and grouped according to body weight since it was difficult to determine their age. Studies were conducted on immature *D. reticulatum* and *C. aspersum,* which weighed 0.5–1.0 g and 1–3 g, respectively.

### 2.2 Maintenance of *M. brunneum* cultures and preparation of conidia

The origin and growth of *M. brunneum* strains ARSEF4556 and V275 on Sabouraud Dextrose Ager (SDA) and broken rice grain is described by Ansari and Butt.^30^ Conidia were harvested from sporulating SDA cultures by gently scraping the surface with a sterile spatula. Conidia were harvested from the surface of rice grain using a MycoHarvester.^31^ Harvested conidia were air dried and weighed before use. Conidia had more than 95% viability as determined by the plate count technique.^32^

### 2.3 Snail avoidance of *M. brunneum* conidia

Choice and no‐choice assays were conducted to see if molluscs avoided *M. brunneum*. In no choice assays, the garden snail was provided lettuce leaves (4 g wet weight) pre‐coated with different doses (0.4, 0.2, 0.04 g) of dry conidia of *M. brunneum* strains V275 and ARSEF4556. Controls consisted of leaves not treated with conidia. Two snails, starved for 24 h, were placed in each test arena 24 h after inoculating the leaves with the fungal inoculum. The assays were performed in a 300 mL cylindrical plastic container at room temperature with leaf consumption based on residual weight being measured 24 and 48 h post‐treatment. There were five replicates per treatment and the whole study repeated twice. Choice assays were as above with the following modifications, the test arena consisted of a 2.5 L ventilated plastic box (16 cm × 10 cm × 16 cm) and lettuce leaves in Petri dishes were placed next to each other with one treated with different doses of *M. brunnuem* conidia and the other left untreated (control). Leaf consumption based on residual weight was measured 24 and 48 h post‐treatment. There were three replicates per treatment and the whole study repeated twice.

### 2.4 Contact toxicity studies

Individual *D. reticulatum* and *C. aspersum* were placed in 9 cm diameter Petri dishes lined with moist Whatman filter paper number 1. The molluscs were exposed to 1, 5 and 10 μL of 1‐octene, 1‐octen‐3‐ol and 3‐octanone, which were applied to the fleshy dorsal surface of the molluscs using an Accumax PRO Micropipette. Control molluscs were left untreated. The Petri dishes were sealed with Parafilm to prevent the molluscs escaping and loss of volatiles. Mortality was recorded 0.5, 1, 3, and 24 h post‐treatment. Slugs and snails were considered dead if they did not respond to probing with a needle.^10^ There were five replicates per treatment with the whole experiment being repeated twice. Assays were conducted at room temperature (21 ± 2 °C).

To determine the mean time to death the above experiments were repeated but mortality was recorded continuously.

### 2.5 Fumigation assay

Individual *D. reticulatum* and *C. aspersum* were exposed to different doses (1, 5 and 10 μL) of 1‐octene, 1‐octen‐3‐ol and 3‐octanone dispensed from a 5 mm diameter filter paper placed on a glass coverslip attached to the lid of a 9 cm diameter Petri dish. Control animals were left untreated. The Petri dishes were sealed with Parafilm to prevent the escape of animals and VOCs. Mortality was recorded 0.5, 1, 3 and 24 h post‐treatment. Slugs and snails were considered dead if they did not respond to probing with a needle.^10^ There were five replicates per treatment with the whole experiment being repeated twice. Assays were conducted at laboratory temperature (21 ± 2 °C).

### 2.6 Repellence assay

**Figure 1:**
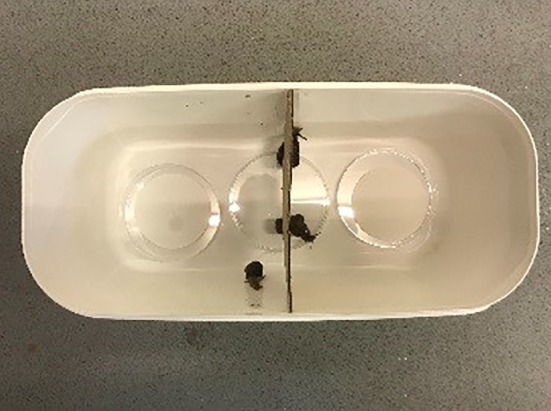
Test arena to evaluate mollusc repellent properties of *Metarhizium brunneum* volatile organic compounds (VOCs). The ventilated plastic container (30 × 13 × 13 cm) was split in two equal halves by a cardboard partition. A hole at the base of the partition allowed free movement of the molluscs. Each chamber contained a Petri dish lined with filter paper with one being treated with different amounts of the active (5, 10, 25, and 50 μL) and the other treated with water only (control). Four animals (*D. reticulatum* or *C. aspersum*) were released from a Petri dish placed in the middle of the arena and their location recorded at 10 min, 0.5, 1, 3, and 24 h post‐treatment.

Repellence of 1‐octene, 1‐octen‐3‐ol and 3‐octanone was investigated using ventilated plastic containers (30 × 13 × 13 cm). Each container was partitioned in the middle by a piece of cardboard with a hole at the base to allow free movement of the animals between the two chambers of the test arena (Fig. 1). A 9 cm diameter Whatman filter paper was placed in each chamber with one paper being treated with different doses (5, 10, 25, 50 μL) of the active and the other with water (control). Four animals (*D. reticulatum, C. aspersum)* were released from a Petri dish placed in the middle of the assay arena and number of animals in each chamber recorded at 10, 30, 60, and 180 min and then every 24 h post‐treatment.

The above study was repeated using *C. aspersum* only. In these assays, 4 g of fresh oilseed rape leaves were placed in each half of the test arena. Four snails starved for 24 h were released in the middle and feeding damage assessed. Leaf damage, based on wet residual weight of the leaves, was determined 24 and 48 h post‐treatment. Both assays were conducted under the same laboratory conditions (21 ± 2 °C).

### 2.7 Antifeedant assay

To determine if leaves exposed to VOCs were avoided by molluscs, 4 g of fresh lettuce leaves were fumigated inside a cylindrical plastic chamber (approximately 6 cm height, 9 cm diameter) lined with a Whatman filter paper. The leaves were exposed to different doses (1, 5, and 10 μL) of 1‐octen‐3‐ol, 3‐octanone or water (control) dispensed from Sharrow King size (7.1 mm diameter) filter tips (Wilsons and Co Ltd, UK). An additional control of no treatment was also included. Exposure to higher doses (>20 μL) caused the leaves to become dehydrated and necrotic. The chamber lid was removed 24 h later, allowing any gases to escape before introducing two healthy snails (starved for 24 h). At this stage VOCs had dispersed and the filter was exhausted. There were five replicates per treatment with the whole study being repeated twice. Snail mortality and leaf damage (based on wet residual weight) were recorded 24 h and 48 h post‐treatment. The study was repeated with the lettuce being replaced with 4 g fresh oilseed rape leaves. Studies were conducted at 21 ± 2 °C.

### 2.8 Evaluation of aqueous formulations of VOCs

Since highly diluted, aqueous formulations of the VOCs may be required to protect crops on a large scale, it was important to establish if these could kill the pest on contact or drive them off the crop. In the first of two studies, individual *D. reticulatum* and *C. aspersum* were exposed to different volumes (10, 20, 50, 100 μL) of 3‐octanone and 1‐octen‐3‐ol diluted in water to 5%, 10%, and 20% (v/v). Water was used as a control. The chemicals were applied using an Accumax PRO Micropipette to the fleshy dorsal surface of the animals. Individuals were placed in a 9 cm diameter Petri dish and sealed with Parafilm. Assays were conducted at room temperature (21 ± 2 °C) and the percentage mortality recorded 0.5, 1, 3, and 24 h post‐treatment. There were five replicates per treatment.

In the second study aqueous formulations were applied to plants infested with the molluscs using a 0.5 L hand‐held sprayer (Screwfix, Loughborough, UK). Briefly, 30 mL of 5% and 20% (v/v) aqueous 1 octen‐3‐ol and 3‐octanone were sprayed onto 25 (30‐day old) corn plants (*Zea mais* variety Madlen, Maisadour Semences, Marsan, France) grown in plastic trays (35 × 20 × 15 cm). Each tray was placed in a Bugdorm. Twenty *D. reticulatum* or *C. aspersum* were released onto the tray a few minutes before spraying. Mortality was recorded 3 and 24 h post‐treatment.

### 2.9 Statistical methods

For *M. brunneum* conidia avoidance studies, the mean and standard error were calculated. Survival was modelled using Generalized Linear Models (GLMs) with logit link functions and binomial error distributions. Since mortality after different time points was observed using different individuals, there was no issue of temporal correlation or censoring typically associated with longitudinal bioassays. Repellence was also modelled as binomial data (repelled *vs*. not repelled). Percentage weight changes were arcsine‐transformed and modelled using Normal linear models. The effects of dose, time post‐treatment, and concentration where included, were modelled as categorical fixed effects and included as main effects and all interactions. In the case of binomial GLMs, over‐dispersion was assessed (and found to be ≪2 in all cases) by comparing residual deviance to residual degrees of freedom. Statistical significance of explanatory variables was tested using likelihood ratio tests assuming a χ^2^ null distribution for binomial GLMs, and F‐ratios for Normal models. All statistical analysis was performed using R v3.5.0.^33^

## 3 RESULTS

### 3.1 Antifeedant properties of *M. bruneum* conidia

**Figure 2:**
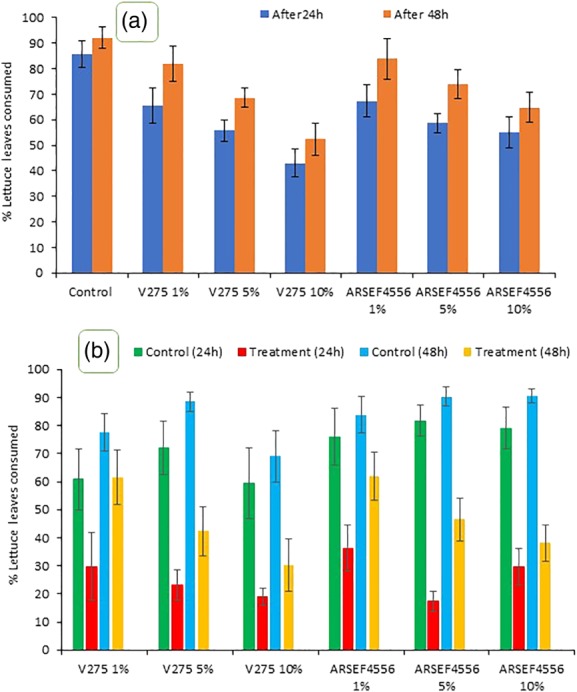
Avoidance behavior of the garden snail to lettuce leaves inoculated with different amounts of conidia of *Metarhizium brunneum* strains V275 and ARESF4556. The amount of food consumed was lower for treated than untreated (control) leaves. Antifeedant activity increased with dose, with conidia of V275 being marginally more repellent. Antifeedant properties of the conidia was generally more pronounced 24 than 48 h post treatment. (a = No choice and b = Choice studies).

In no choice assays, *Cornu aspersum* consumption of lettuce leaves treated with conidia of *M. brunneum* was dependent upon the fungal dose and strain (Fig. 2(a)). It was more pronounced for strain V275 especially at the highest dose. The percentage lettuce leaf consumed was lower 24 h than 48 h post‐treatment for both strains (Fig. 2). In choice assays, the snails tended to move towards the treated leaves before moving to the untreated leaves. This was particularly obvious at the low doses of both strains and in the first hour of the experiment. The snails spent only a few minutes at the treated leaf with insignificant feeding before moving towards the untreated leaf. The snails consumed most of the untreated leaf material before returning to the treated leaf or wandered in the test arena. Leaf consumption of treated leaves was linked with dose, strain and time (Fig. 2(b)). Far less was consumed 24 h than 48 h post‐treatment and consumption was generally lower for leaves treated with conidia of V275 (Fig. 2(b)).

### 3.2 Contact toxicity assays

**Figure 3:**
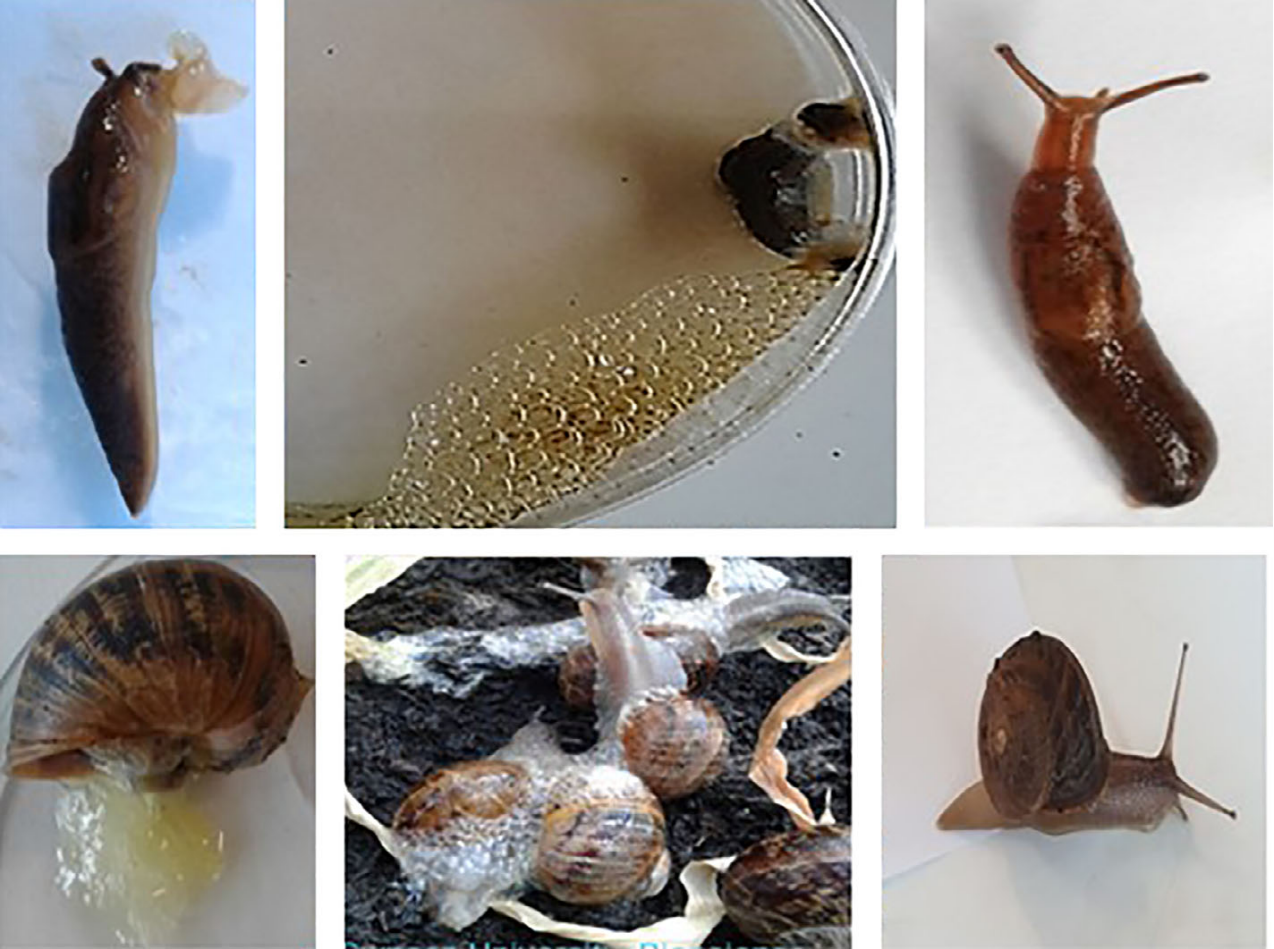
Behavior of slugs and snails when exposed to the volatile organic compounds (VOCs) 1‐octene, 3‐octanone and 1‐octen‐3‐ol. Slugs occasionally vomited a brownish fluid (top left) or produced a brownish froth which readily stained the underlying moist filter paper (top middle). Dead or dying animals were immobile and appeared shriveled (top left and middle) unlike the healthy slug (top right). Snails exposed to VOCs produced either pale yellow (bottom left) or clear frothy secretions (bottom middle) unlike the healthy control snails (bottom right).

Our attention focused on the ubiquitous fungal VOCs, 1‐octene, 1‐octen‐3‐ol and 3‐octanone. These compounds were toxic on contact, causing 100% mortality of adult and juvenile slugs and snails including *Cornu aspersum*, *Limax maximus*, *Arion ater*, *Arion rufus*, *Derocerus reticulatum* and *Limax flavus* (data not presented). In‐depth studies conducted on *D. reticulatum* and *C. aspersum* showed that they exhibited various behaviors depending on the chemical, dose and method of exposure. Slugs exposed to the VOCs vomited a brown liquid or exhibited contorted body movements, producing yellow or brown staining mucus before shrinking and dying (Fig. 3). Snails exposed to the VOCs withdrew into the shell or produced a yellow watery mucus before dying often with their head exposed. Treating snails with diluted aqueous formulations triggered production of clear, frothy mucus (Fig. 3).

**Figure 4:**
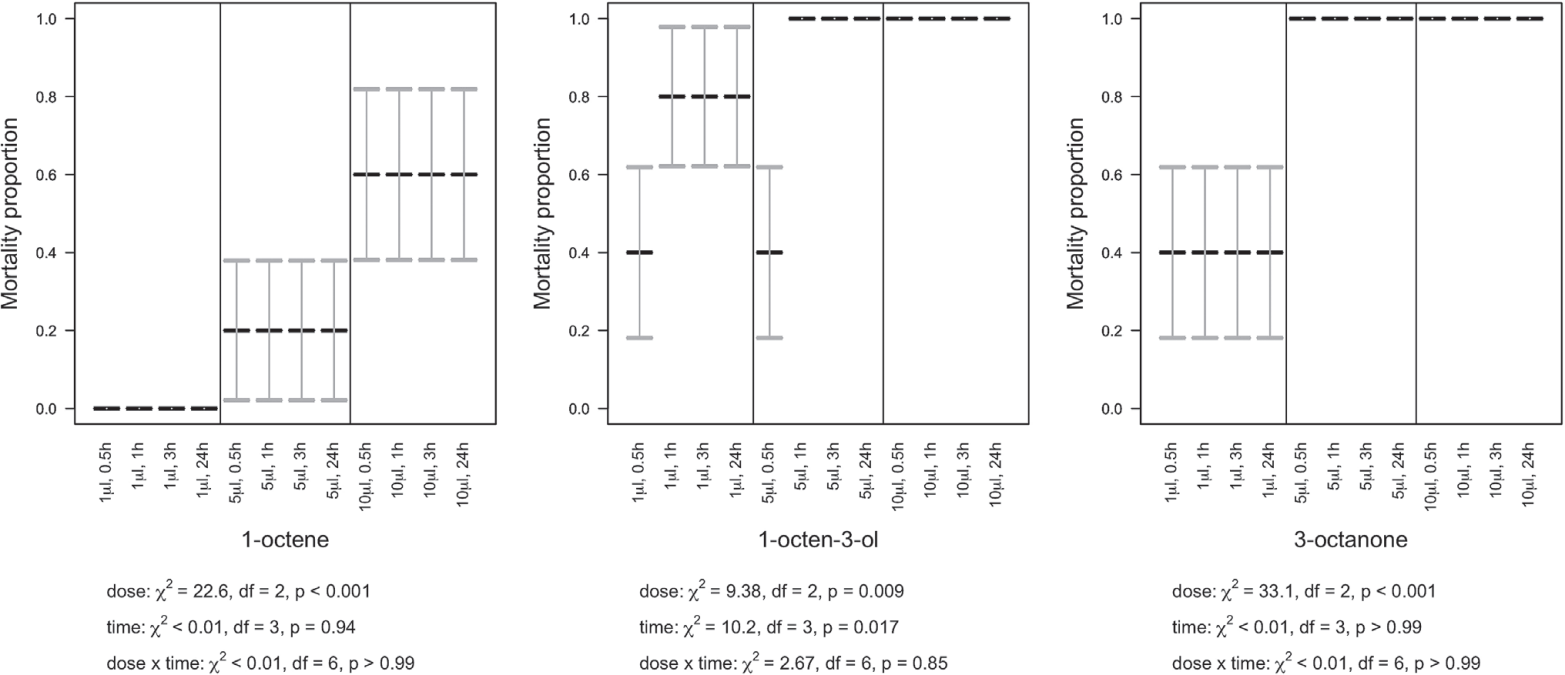
Volatile organic compound (VOC) contact effect on survival of the slug *D. reticulatum*. Slug mortality (means with SE error bars) over 24 h, under varying concentrations of 1‐octene, 1‐octen‐3‐ol, and 3‐octanone.

**Figure 5:**
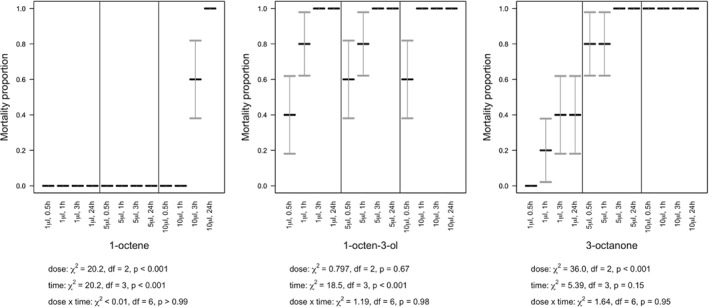
Volatile organic compound (VOC) contact effect on survival of the snail, *C. aspersum*. Snail mortality (means with SE error bars) over 24 h, under varying concentrations of 1‐octene, 1‐octen‐3‐ol, and 3‐octanone.

Mortality of *D. reticulatum* and *C. aspersum* was dependent upon the VOC, dose and time post‐treatment (Table 1, Figs. 4 and 5). The statistical interaction between dose and time post‐treatment was not significant for either species (slugs: *P* > 0.85 for all VOCs; snails: *P* > 0.95 for all VOCs), with any of the three VOCs. Overall, mortality generally increased with increasing dose (slugs: 1‐octene, χ^2^_2_ = 22.6, *P* < 0.001, 1‐octen‐3‐ol, χ^2^_2_ = 9.38, *P* = 0.009, 3‐octanone, χ^2^_2_ = 33.1, *P* < 0.001; snails: 1‐octene, χ^2^_2_ = 20.6, *P* < 0.001, 1‐octen‐3‐ol, χ^2^_2_ = 0.797, *P* = 0.67, 3‐octanone, χ^2^_2_ = 36.0, *P* < 0.001) and time (slugs: 1‐octene, χ^2^_3_ < 0.01, *P* > 0.99, 1‐octen‐3‐ol, χ^2^_3_ = 10.2, *P* = 0.017, 3‐octanone, χ^2^_3_ < 0.01, *P* > 0.99; snails: 1‐octene, χ^2^_3_ = 20.6, *P* < 0.001, 1‐octen‐3‐ol, χ^2^_3_ = 18.5, *P* < 0.001, 3‐octanone, χ^2^_3_ = 5.39, *P* = 0.15), but mortality was higher with 1‐octen‐3‐ol or 3‐octanone than with 1‐octene.

**Table 1:** Time to death of garden slug *D. reticulatum* (weight 0.5–2 g) and garden snail *C. aspersum* (weight 1–3 g) exposed to different volumes of 1‐octene, 1‐octen‐3‐ol and 3‐octanone. The table also shows corresponding percentage morality at the time of death

		*D. reticulatum*		*C. aspersum*	
VOCs	Doses (μL)	Time to death (min) ± SE	Mortality (%) ± SE	Time to death (min) ± SE	Mortality (%) ± SE
1‐octene	1	All Alive	0	All Alive	0
5	516.5 ± 302.6	40 ± 24.5	All Alive	0
10	252.4 ± 76.2	100	247.6 ± 121.3	100
1‐octen‐3ol	1	79.2 ± 3	100	42.2 ± 9.2	100
5	42 ± 10.1	100	37 ± 9.6	100
10	29.6 ± 0	100	27.4 ± 6.2	100
3‐octanone	1	43 ± 9.7	20 ± 20	55.5 ± 10.4	40 ± 25.8
5	37 ± 4.7	100	36 ± 17.4	100
10	31.2 ± 8.9	100	10.2 ± 0.7	100
Control	0	All Alive	0	All Alive	0

### 3.3 Fumigation assays

**Figure 6:**
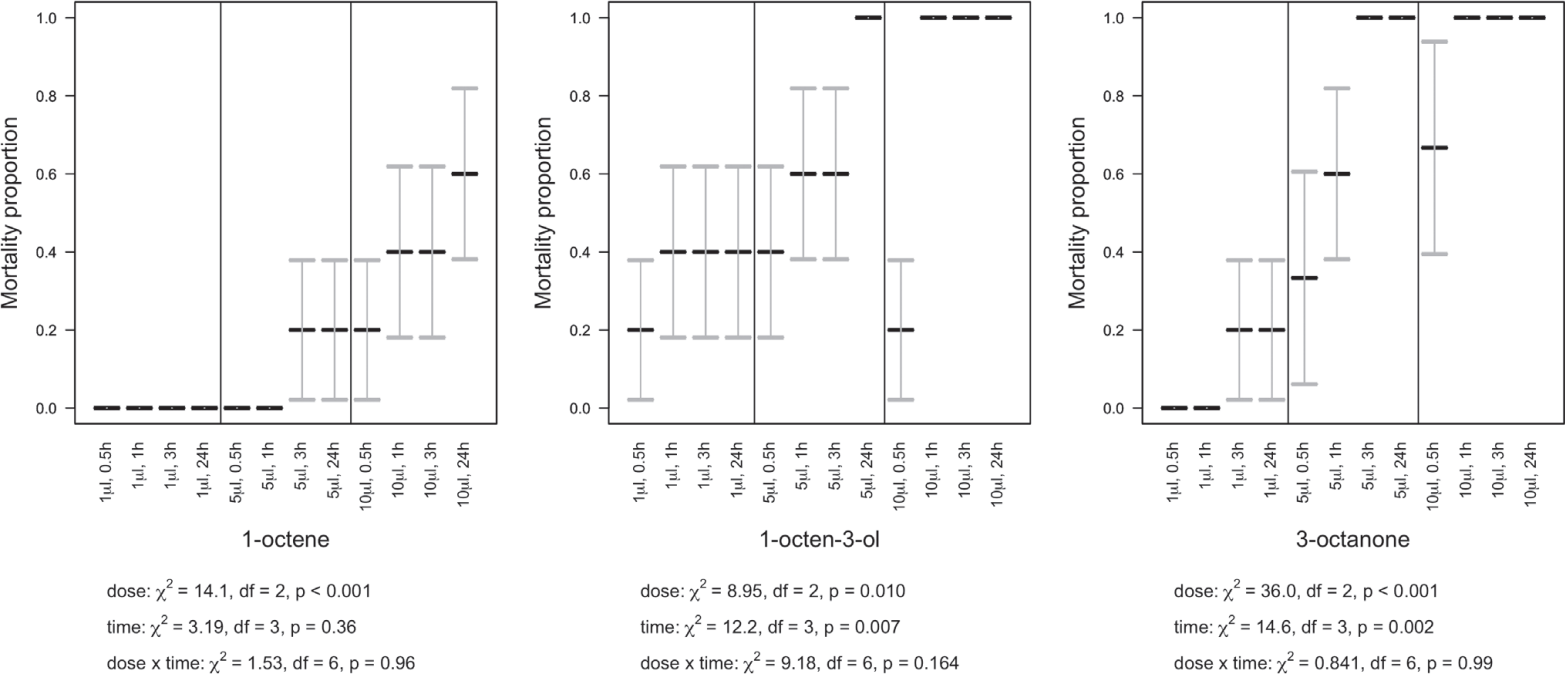
Volatile organic compound (VOC) fumigation effect on survival of the slug *D. reticulatum*. Slug mortality (means with SE error bars) over 24 h, under varying concentrations of 1‐octene, 1‐octen‐3‐ol, and 3‐octanone.

**Figure 7:**
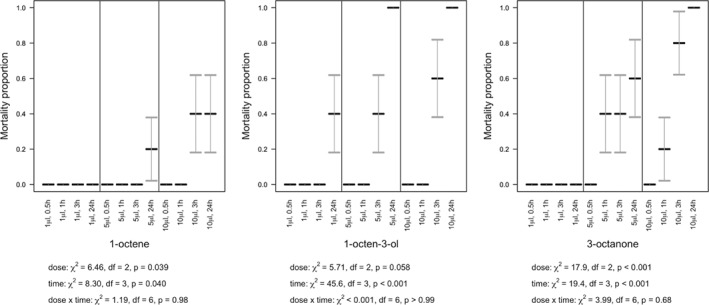
Volatile organic compound (VOC) fumigation effect on survival of the snail, *C. aspersum*. Snail mortality (means with SE error bars) over 24 h, under varying concentrations of 1‐octene, 1‐octen‐3‐ol, and 3‐octanone.

Fumigation of slugs and snails also proved lethal (Figs. 6 and 7) but marginally less potent than direct contact. As with direct contact, the statistical interaction between dose and time post‐treatment was not significant for either species (slugs: *P* > 0.16 for all VOCs; snails: *P* > 0.68 for all VOCs), with any of the three VOCs. Similarly, there were typically increases in mortality with both dose (slugs: 1‐octene, χ^2^_2_ = 14.1, *P* < 0.001, 1‐octen‐3‐ol, χ^2^_2_ = 8.95, *P* = 0.010, 3‐octanone, χ^2^_2_ = 36.0, *P* < 0.001; snails: 1‐octene, χ^2^_2_ = 6.46, *P* = 0.039, 1‐octen‐3‐ol, χ^2^_2_ = 5.71, *P* = 0.058, 3‐octanone, χ^2^_2_ = 17.9, *P* < 0.001) and time (slugs: 1‐octene, χ^2^_3_ = 3.19, *P* > 0.36, 1‐octen‐3‐ol, χ^2^_3_ = 12.2, *P* = 0.007, 3‐octanone, χ^2^_3_ = 14.6, *P* = 0.002; snails: 1‐octene, χ^2^_3_ = 8.30, *P* = 0.040, 1‐octen‐3‐ol, χ^2^_3_ = 45.6, *P* < 0.001, 3‐octanone, χ^2^_3_ = 19.4, *P* < 0.001), and mortality was higher with 1‐octen‐3‐ol or 3‐octanone than with 1‐octene.

### 3.4 Repellence assays

**Figure 8:**
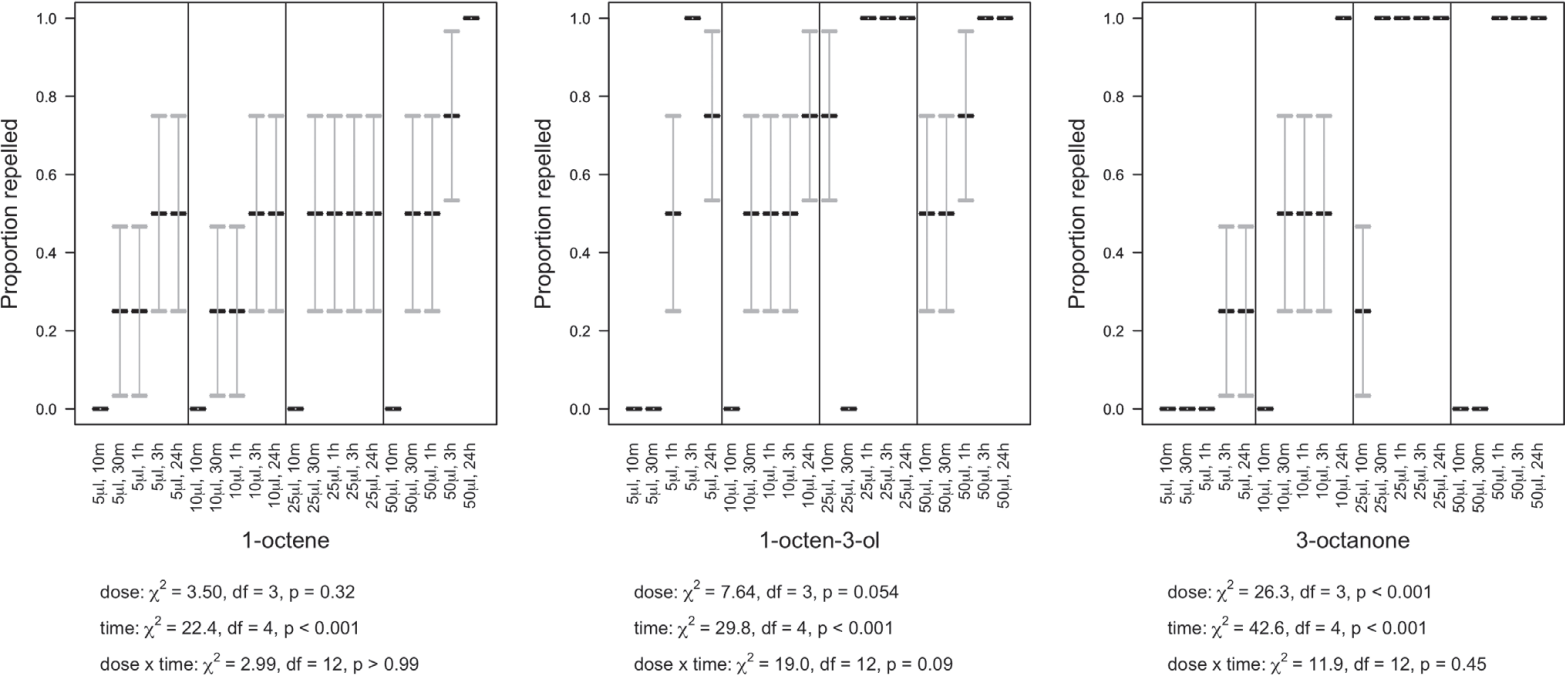
Slug repellence (means with SE error bars) over 24 h, under varying concentrations of 1‐octene, 1‐octen‐3‐ol, and 3‐octanone.

Slugs were repelled by 1‐octene but the statistical interaction between dose and time post‐treatment was not significant (χ^2^_df = 12_ = 2.99, *P* > 0.99) (Fig. 8). However, repellence increased with increasing time (χ^2^_df = 4_ = 22.4, *P* < 0.001) but not dose (χ^2^_df = 3_ = 3.50, *P* = 0.32) (Fig. 8). Where 1‐octen‐3‐ol was applied, dose was not found to be statistically significant, either in its interaction with time (χ^2^_df = 12_ = 19.02, *P* = 0.09) or as a main effect (χ^2^_df = 3_ = 7.64, *P* = 0.054) but mortality significantly increased with time post‐treatment (χ^2^_df = 4_ = 29.8, *P* < 0.001) (Fig. 8). Where 3‐octanone was applied, the statistical interaction between dose and time post‐treatment was not significant (χ^2^_df = 12_ = 11.9, *P* = 0.45) but repellence increased with both time (χ^2^_df = 4_ = 42.6, *P* < 0.001) and dose (χ^2^_df = 3_ = 26.3, *P* < 0.001) (Fig. 8).

**Figure 9:**
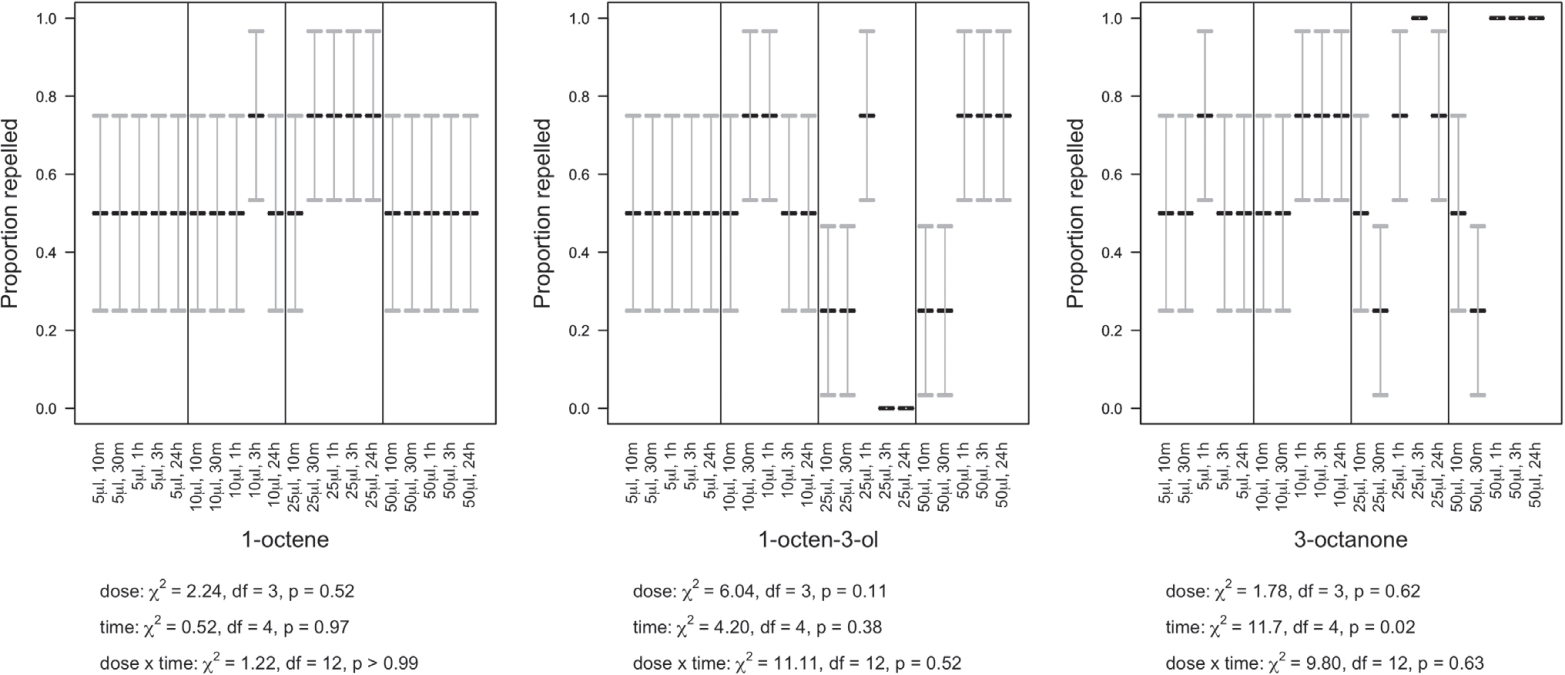
Snail repellence (means with SE error bars) over 24 h, under varying concentrations of 1‐octene, 1‐octen‐3‐ol, and 3‐octanone.

Snails were repelled by 1‐octene but the statistical interaction between dose and time post‐treatment was not significant (χ^2^_df = 12_ = 1.22, *P* > 0.99) (Fig. 9). Furthermore, neither time (χ^2^_df = 4_ = 0.52, *P* = 0.97) nor dose (χ^2^_df = 3_ = 2.24, *P* = 0.52) increased repellence as a main effect. This finding was repeated with 1‐octen‐3‐ol: the statistical interaction between dose and time post‐treatment was not significant (χ^2^_df = 12_ = 11.11, *P* = 0.52), nor time (χ^2^_df = 4_ = 4.20, *P* = 0.38) or dose (χ^2^_df = 3_ = 6.04, *P* = 0.11) as a main effect (Fig. 9). Where 3‐octanone was applied, dose was not found to be statistically significant, either in its interaction with time (χ^2^_df = 12_ = 9.80, *P* = 0.63) or as a main effect (χ^2^_df = 3_ = 1.78, *P* = 0.62). However, repellence did increase with increasing time after treatment (χ^2^_df = 4_ = 11.7, *P* = 0.02) (Fig. 9).

### 3.5 Antifeedant assays

**Figure 10:**
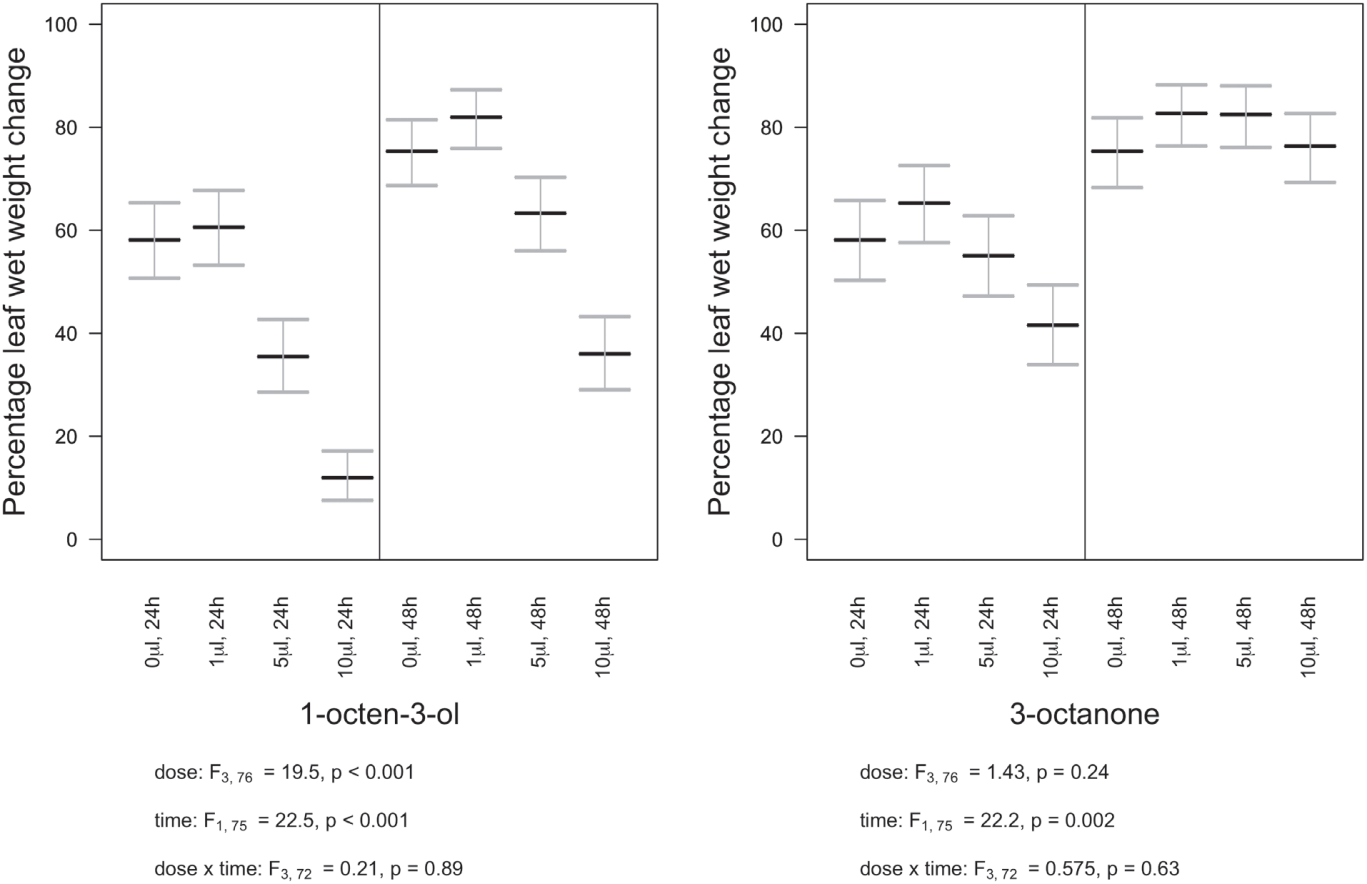
Snail antifeedant properties of 1‐octen‐3‐ol and 3‐octanone as reflected in % wet weight change of fresh leaves. Weight change (means with SE error bars) over 48 h, under varying concentrations of 1‐ octen‐3‐ol and 3‐octanone.

Snail feeding damage was reduced if leaves were fumigated with VOCs. With 1‐octen‐3‐ol the statistical interaction between dose and time post‐treatment was non‐significant (F_3,72_ = 0.21, *P* = 0.89). Leaf weight change increased with time (F_1,75_ = 22.5, *P* < 0.001) but decreased with dose (F_3,76_ = 19.5, *P* < 0.001) (Fig. 10). Where 3‐octanone was applied, dose was not found to be statistically significant, either in its interaction with time (F_3,72_ = 0.575, *P* = 0.63) or as a main effect (F_3,76_ = 1.43, *P* = 0.24). However, weight change did increase with increasing time after treatment (F_1,75_ = 22.2, *P* < 0.002) (Fig. 10). It should be noted, however, that snails survived all treatments except at the highest dose (10 μL) of 1‐octen‐3‐ol which resulted in 20% mortality 24 h post‐treatment.

### 3.6 Aqueous formulation assays

**Figure 11:**
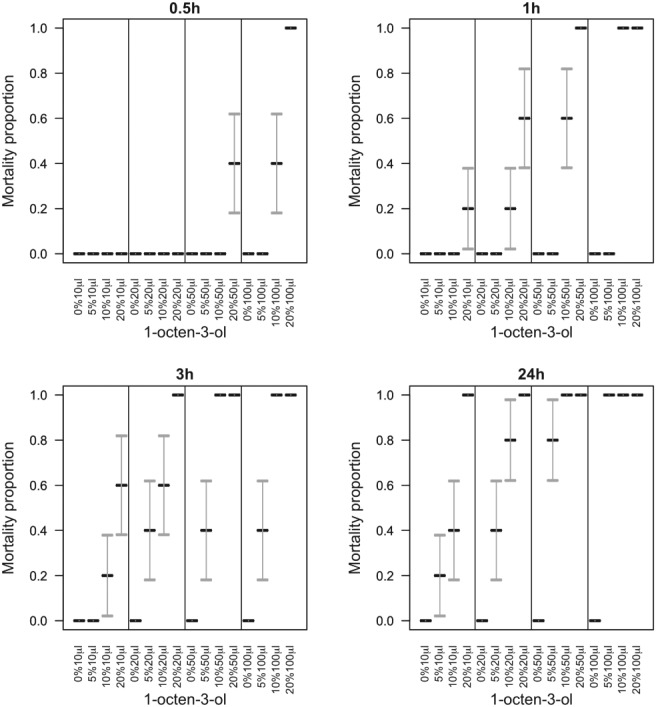
Evaluation of different volumes of aqueous formulations of 1‐octen‐3‐ol applied to the slug mantle. Slug mortality (means with SE error bars) over 24 h, under varying concentrations of 1‐octen‐3‐ol. None of the statistical interactions between dose, time post‐treatment, and concentration were significant (χ^2^ = 23.0, df = 54, *P* > 0.99) but mortality increased with dose (χ^2^ = 80.1, df = 3, *P* < 0.001), time (χ^2^ = 114, df = 3, *P* < 0.001), and concentration (χ^2^ = 207, df = 3, *P* < 0.001).

**Figure 12:**
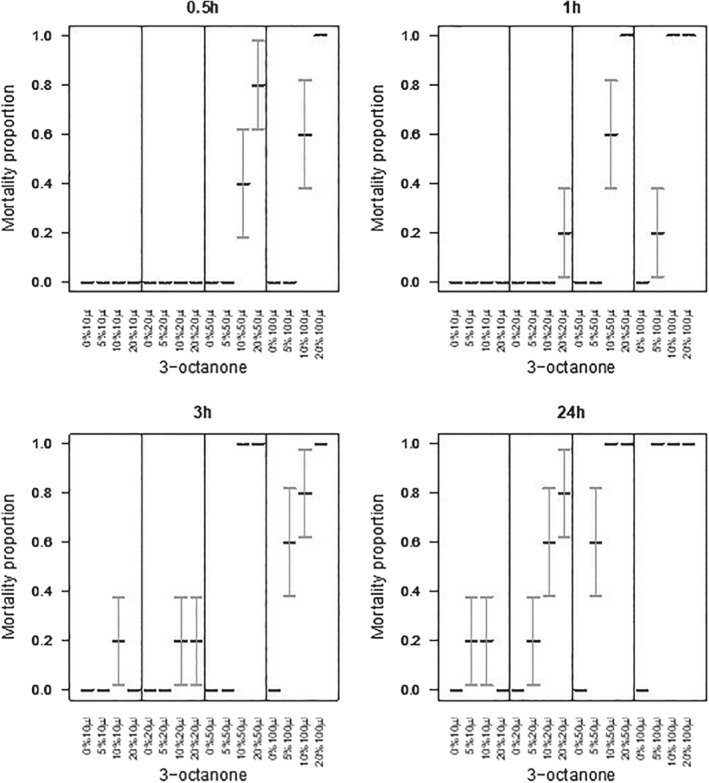
Evaluation of different volumes of aqueous formulations of 3‐octanone applied to slug mantle. Slug mortality (means with SE error bars) over 24 h, under varying concentrations of 3‐octanone. The three‐way interaction between dose, time, and concentration was not significant (χ^2^ < 0.001, df = 27, *P* > 0.99). The only significant two‐way interaction was a synergistic effect on mortality between dose and concentration (χ^2^ = 19.2, df = 9, *P* = 0.023) (Fig. 12). As main effects, mortality increased with dose (χ^2^ = 143, df = 3, *P* < 0.001), time (χ^2^ = 51.5, df = 3, *P* < 0.001), and concentration (χ^2^ = 158, df = 3, *P* < 0.001).

Application of aqueous formulations of the VOCs to slugs showed that where the active was 1‐octen‐3‐ol, none of the statistical interactions between dose, time post‐treatment, and concentration were significant (χ^2^_df = 54_ = 23.0, *P* > 0.99) but mortality increased with dose (χ^2^_df = 3_ = 80.1, *P* < 0.001), time (χ^2^_df = 3_ = 114, *P* < 0.001), and concentration (χ^2^_df = 3_ = 207, *P* < 0.001) (Fig. 11). Where 3‐octanone was applied, the three‐way interaction between dose, time, and concentration was not significant (χ^2^_df = 27_ < 0.001, *P* > 0.99) (Fig. 12). The only significant two‐way interaction was a synergistic effect on mortality between dose and concentration (χ^2^_df = 9_ = 19.2, *P* = 0.023) (Fig. 12). As main effects, mortality increased with dose (χ^2^_df = 3_ = 143, *P* < 0.001), time (χ^2^_df = 3_ = 51.5, *P* < 0.001), and concentration (χ^2^_df = 3_ = 158, *P* < 0.001) (Fig. 12).

**Figure 13:**
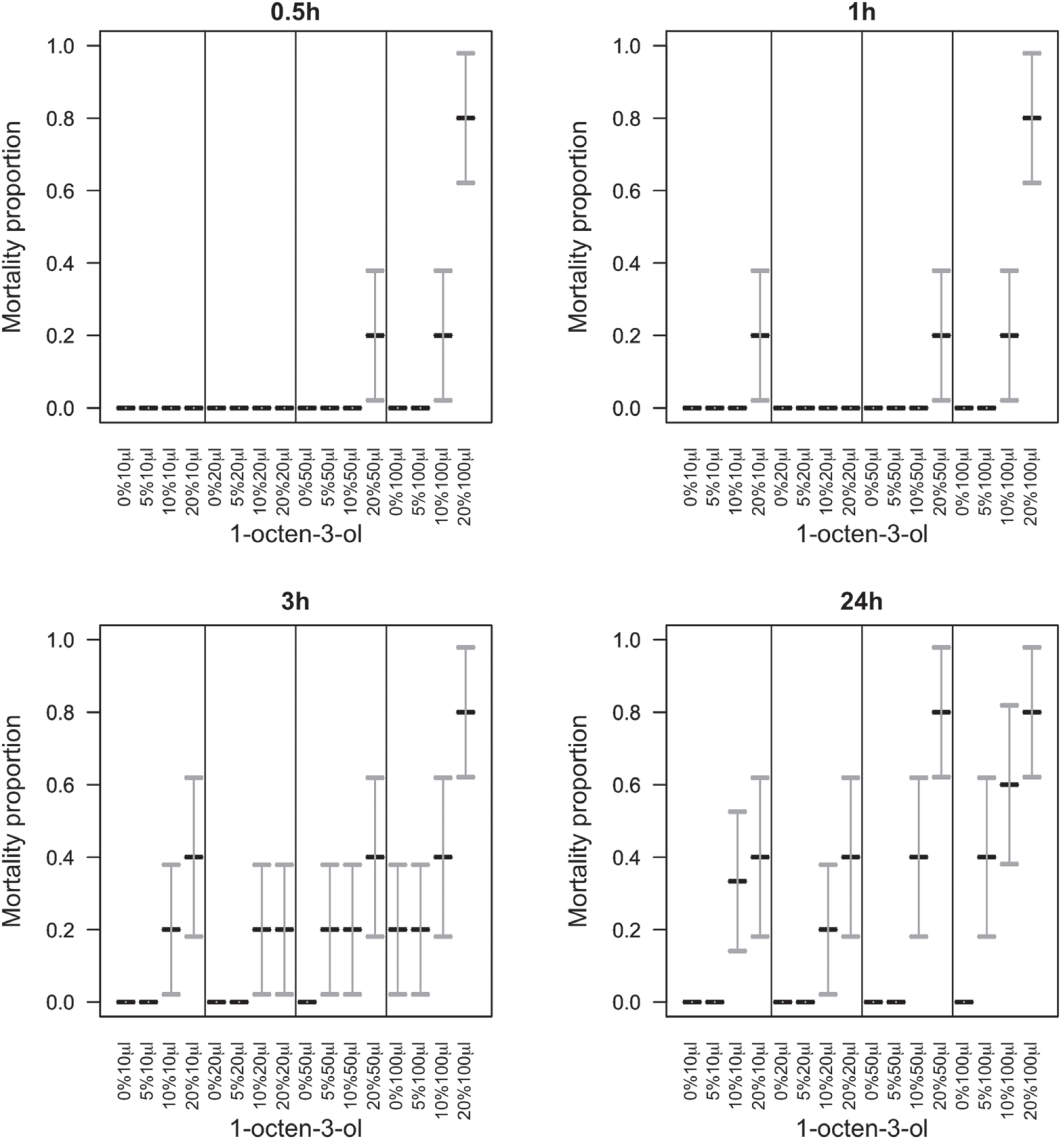
Evaluation of different volumes of aqueous formulations of 1‐octen‐3‐ol applied to snail mantle. Snail mortality (means with SE error bars) over 24 h, under varying concentrations of 1‐octen‐3‐ol. None of the statistical interactions between dose, time post‐treatment, and concentration were significant (χ^2^df = 54 = 20.3, *P* > 0.99) but mortality increased with dose (χ^2^ = 35.6, df = 3, *P* < 0.001), time (χ^2^ = 24.8, df = 3, *P* < 0.001), and concentration (χ^2^ = 69.2, df = 3, *P* < 0.001).

**Figure 14:**
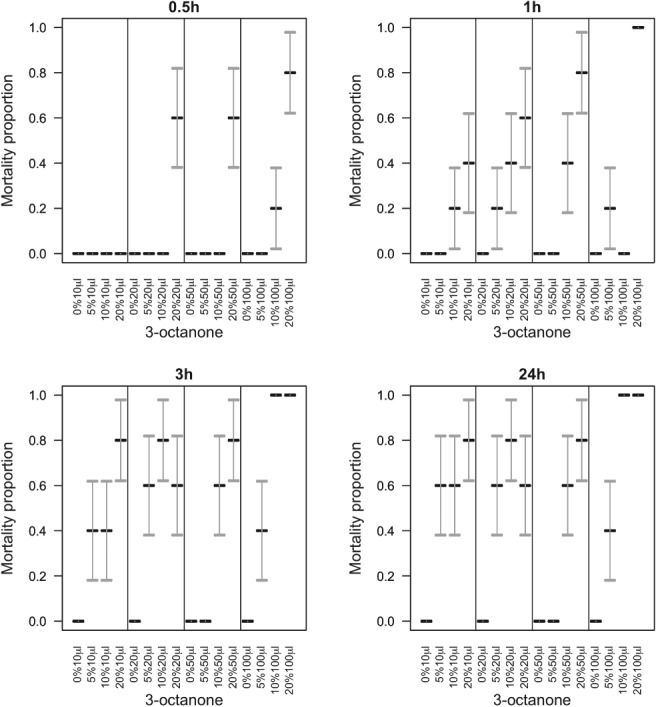
Contact toxicity of different volumes of aqueous formulations of 3‐octanone applied to snail mantle. Snail mortality (means with SE error bars) recorded over 24 h, under varying concentrations of 3‐octanone. The three‐way interaction between dose, time, and concentration was not significant (χ^2^ = 13.1, df = 27, *P* = 0.99). The only significant two‐way interaction was a synergistic effect on mortality between dose and concentration (χ^2^ = 20.8, df = 9, *P* = 0.014). As main effects, mortality increased with dose (χ^2^ = 12.1, df = 3, *P* = 0.007), time (χ^2^ = 48.7, df = 3, *P* < 0.001), and concentration (χ^2^ = 138, df = 3, *P* < 0.001).

Application of aqueous formulations of VOCs to snails showed that with 1‐octen‐3‐ol none of the statistical interactions between dose, time post‐treatment, and concentration were significant (χ^2^_df = 54_ = 20.3, *P* > 0.99) but mortality increased with dose (χ^2^_df = 3_ = 35.6, *P* < 0.001), time (χ^2^_df = 3_ = 24.8, *P* < 0.001), and concentration (χ^2^_df = 3_ = 69.2, *P* < 0.001) (Fig. 13). Where 3‐octanone was applied, the three‐way interaction between dose, time, and concentration was not significant (χ^2^_df = 27_ = 13.1, *P* = 0.99) (Fig. 14). The only significant two‐way interaction was a synergistic effect on mortality between dose and concentration (χ^2^_df = 9_ = 20.8, *P* = 0.014). As main effects, mortality increased with dose (χ^2^_df = 3_ = 12.1, *P* = 0.007), time (χ^2^_df = 3_ = 48.7, *P* < 0.001), and concentration (χ^2^_df = 3_ = 138, *P* < 0.001) (Fig. 14).

## 4 DISCUSSION

This is the first study to show that conidia of insect pathogenic fungi possess mollusc antifeeding properties. The fact that conidia of *M. brunneum* reduced feeding of starved snails suggests that the spores possess irritants or antifeedant compounds that protect against grazing by fungivores in the soil, the natural habitat of this fungus.^34^ Most surprising was the movement of the snails to the treated leaves, which suggest the spores were also emitting an attractant. We postulate that the attractants helped with spore dispersal as reported for collembola, which respond to 1‐octen‐3‐ol and other VOCs produced by *Metarhizium* conidia.^35^ *Metarhizium* infected insects produce copious conidia at the surface of the mycosed cadaver, which would result in a concentration of the antifeedant or irritant compounds and may explain why there are no reports of molluscs feeding on mycosed cadavers. Conidia of *M. brunneum* strain V275 appeared to have greater antifeedant or irritant properties suggesting that the type and quantity of irritant/antifeedant compounds vary between strains. It is possible that the irritant/antifeedant compounds are labile since the snails returned to the treated leaves after they had consumed the untreated leaves.

Although *Metarhizium* species produce a wide range of VOCs,^24^, ^29^ the eight‐carbon volatiles, 1‐octene, 1‐octen‐3‐ol and 3‐octenone, are of particular interest because these ubiquitous fungal compounds which are known to play a major role in fungal‐invertebrate interactions.^23^, ^36^ These VOCs have been shown to influence the behavior of a wide range of insects, acting as either attractants or repellents.^22^, ^37^ One notable study is that of Yanagawa *et al*.,^28^ who reported that termite aversion of the insect pathogenic fungus *Isaria fumosorosea* Wize was due to 3‐octanone and 1‐octen‐3‐ol present in small amounts (0.01 ng per 10^7^ conidia) on the conidial surface. At such small quantities, these compounds may not repel molluscs but may act as irritants or antifeedants. If this is the case, then it would explain why the snails in the current study avoided feeding on leaves treated with *M. brunneum* conidia.

Very few VOCs have been shown to have pesticidal properties. The current study is the first to show that the fungal VOCs 1‐octene, 1‐octen‐3‐ol and 3‐octenone are toxic to the terrestrial molluscs *D. reticulatum* and *C. aspersum* on contact or when deployed as fumigants. Both *D. reticulatum* and *C. aspersum* try to avoid direct exposure or food tainted by these compounds. Both molluscs produced secretions in response to the volatiles, presumably as part of a defence and repair response.^38^ The secretions could also be an innate stress response to neutralize the fungal VOCs or prevent secondary infections *via* damaged tissue since the mucus is supposed to have antimicrobial properties.^39^

The VOCs of *Metarhizium* and other fungi are repellents for several invertebrates including store grain pests (*Sitophilus zeamais Motschulsky)*, and termites (*Coptotermes formosanus* Shiraki).^21^, ^27^, ^28^ Repellency is dependent upon the target since some insects are tolerant of higher quantities than others. For example, mushroom pests such as the phorid fly, *Megaselia halterata* Wood, are tolerant of relatively high levels of 1‐octen‐3‐ol.^40^ In the current study, the VOCs killed both *D. reticulatum* and *C. aspersum* on contact even at relatively low doses. Based on their behaviour and time to death the compounds were toxic irritants. The snail was more tolerant than the slug in the fumigation assays, presumably because it could withdraw into the impervious shell.

Both *D. reticulatum* and *C. aspersum* avoided 1‐octen‐3‐ol and 3‐octenone environments including food exposed to these compounds, presumably the VOCs inform the pests of food quality since many molds producing these VOCs also produce harmful toxins. Gebauer^41^ recorded high mortality in *D. reticulatum* fed wheat grain contaminated with *Fusarium*, a ubiquitous toxigenic fungus. Avoidance behavior has been reported for beetle and weevil pests of stored grain^21^ and parasitoids of the grain beetle larvae.^42^ For example, females of the parasitoid *Lariophagus distinguendus* Forster are strongly attracted to the odor of the feces of the granary weevil (*Sitophilus granaries Linnaeus)* larval host but avoid feces which has absorbed 1‐octen‐3‐ol emitted by the toxigenic grain mold *Aspergillus* since this reduces host fitness.^42^

Entomopathogenic fungi belonging to the genera *Beauveria* and *Metarhizium* are known to produce VOCs that influence the behavior of many insect species.^20^ Most insects exhibit avoidance behavior, presumably to avoid infection since avoidance is often linked to virulence.^29^, ^43^, ^44^ Repellency has been reported for termites, seven‐spot ladybirds (*Coccinella septempunctata Linnaeus)* and poultry red mites (*Demanysussus gallinae* deGeer).^29^, ^44^, ^45^ Volatiles emitted by inoculum of *Metarhizium* species may account for the ovipositional deterrence behavior of several insects including sweet potato weevil (*Cylas formicarius* Fabricius), house flies (*Musca domestica* Linneaus) and stable flies (*Stomoxys calcitrans* Linneaus).^43^, ^46^ Presumably, adults recognize environments that may be hostile to the eggs or emergent larvae since *Metarhizium* is capable of infecting both eggs and larvae.^47^ In contrast, very little is known about the susceptibility of slugs and snails to fungal infection. Laboratory studies show that entomopathogenic and nematophagous fungi are able to infect molluscan eggs. Ovicidal activity has been reported for the aquatic snail, *Biomphalaria glabrata* Say, inoculated with *M. anisopliae* (Metchnikoff) Sorokin and the grey field slug, *Agriolimax agrestis* Linneaus, infected by *Pochonia chlamydosporia* Goddard.^48^, ^49^ It is possible that molluscs use the VOC cues to avoid sites where pathogenic fungi are present to ensure their eggs are not attacked.

Application of aqueous formulations of 1‐octen‐3‐ol and 3‐octanone complemented studies using neat compounds. They showed that mortality was dose‐related and that slugs were more susceptible to the VOCs than the snail. The fact that significant mortality was observed 24 h post‐treatment suggests that either the VOCs continue to cause damage or substantial irreparable damage from which the animal could not recover. Indeed, fungal VOCs are known to have neurotoxic or cytotoxic properties with some of the toxicity mediated through the generation of reactive oxygen species.^50^ The VOCs have much potential as replacement products for metaldehyde with many ecological benefits. For example, the natural VOCs are ephemeral so leave no residues. In a crop, the VOCs would deter molluscs entering the treated area. Exactly how long the repellency would last would depend on the formulation and whether the molluscs could be conditioned to avoid VOC‐treated crops. Molluscs are supposed to have the ability to acquire a long‐term olfactory memory after a single conditioning session.^51^ To ensure the molluscs do not migrate back to the treated crop, it would be imperative to include a trap crop. This ‘push‐pull’ strategy would allow for more targeted control of the pest with concomitant reduced pesticide input. Another commercial use of the VOCs would be as fumigants, to treat produce which may be infested with exotic pests such as golden apple snails.^7^ Ephemeral fungal VOCs would pose less of a risk than conventional chemical molluscicides to non‐target animals including predators and decomposers.^52^
